# Overview of the Use of Optical Coherence Tomography Angiography in Neovascular Age-Related Macular Degeneration

**DOI:** 10.3390/jcm13175042

**Published:** 2024-08-25

**Authors:** Henrik Faatz, Albrecht Lommatzsch

**Affiliations:** 1 Eye Center, St. Franziskus Hospital Münster, 48145 Münster, Germany; albrecht.lommatzsch@augen-franziskus.de; 2 Achim Wessing Institute for Imaging in Ophthalmology, University of Essen-Duisburg, 45147 Essen, Germany; 3 Department of Ophthalmology, University of Essen-Duisburg, 45147 Essen, Germany

**Keywords:** retina, imaging, AMD, octa

## Abstract

The aim of this review is to present and discuss the use of optical coherence tomography angiography (OCTA) in age-related macular degeneration (AMD). OCTA is a non-invasive imaging procedure that gives a detailed indirect view of physiological and pathological vessels in the retina and choroid membrane. Compared with dye-based imaging, OCTA provides a segmented presentation of the individual vascular layers and plexuses, thus enabling previously unattainable differentiation and classification of pathological vascular changes within or underneath the retina. In particular, OCTA facilitates early detection of exudative macular neovascularizations (MNV) so that treatment with anti-VEGF medication can be initiated. Moreover, in the context of both screening and therapy monitoring, it is hoped that OCTA can provide more detailed data to enable greater personalization of treatment and follow-up. The image quality of OCTA is, however, susceptible to artifacts, and validation of the results by studies is required. Recent developments have shown constant improvement both in the algorithms for image calculation and avoidance of artifacts and in image quality, so the scope of OCTA will certainly expand with time.

## 1. Introduction

Ever since its first description in 2010, optical coherence tomography angiography (OCTA) has attracted increasing interest from researchers, reflected in constant growth in publications on its use in ophthalmology [1]. The principal advantages of OCTA over dye-based imaging are the following:

- It is a non-invasive procedure with practically no local or systemic adverse effects; at worst, allergic shock;
- Less severe dazzling;
- Shorter examination time; and
- Reliable reproducibility.

To achieve high scanning speed, spectral domain (SD) or swept source (SS) are used. SS-OCTA uses a wavelength in the infrared range of 1050 nm. One advantage compared to SD-OCTA, which uses a wavelength of 800–870 nm, is therefore the greater penetration depth, allowing for better visualization of deeper structures such as the choroid and sclera [2]. The mathematical algorithms for calculating these differences vary among manufacturers. A phase-based, amplitude-based, or combined algorithm is applied. The resulting volume of the retinal vessels is then segmented into individual layers. This segmentation is automatically generated by the device software and is based on the hyper-reflective boundaries of the retinal layers. Especially in macular diseases, the anatomical layers are disrupted by intraretinal and subretinal hemorrhages or PEDs, preventing the device software from correctly detecting the layer boundaries [3]. Incorrect segmentation leads to artifacts, so it is essential to examine the corresponding B-scan during the analysis of OCTA images. This ensures that the segmentation lines are accurately represented, providing correct orientation within the retinal layers. The user can correct and adjust the segmentation lines and thus change the area to be examined. One of the current disadvantages of OCTA is that light reflections at hyper-reflective interfaces within the retina, such as the RPE, can cause projection artifacts of retinal vessels. These projection artifacts can be eliminated using algorithms, with the corresponding areas shown in black in the image. However, important information can also be lost if the structure of interest is overlaid by a projection artifact. Additional artifacts can arise from patient eye movements during the scan, leading to horizontal and vertical bands on the images, rendering these areas unassessable. While eye-tracking systems and fast acquisition times are employed to prevent these artifacts, algorithms can also be used to correct them retrospectively. However, this can introduce new artifacts or result in loss of information [4]. It is essential to be aware of all possible artifacts to avoid misdiagnosis when evaluating OCTA images.

Depending on the device manufacturer, the software offers a range of quantification tools to evaluate the acquired images. This provides the user with the ability to measure distances and areas, as well as to display the average flow in selected regions, reflecting the proportion of detected vessels. It is also possible to automatically calculate the size of non-perfused areas, such as the foveal avascular zone. An automated algorithm for detecting an MNV or classifying it as active or inactive is not yet possible, but it would be a desirable goal for the future.

It would therefore be advantageous if OCTA could be established as a routine means of vascular visualization for purposes of diagnosis and therapeutic decision-making in retinal vascular pathology. Constant refinement of the image calculation software for OCTA devices has reduced the initial problems with image quality, particularly with regard to artifacts, so larger images can be generated. Nevertheless, the application of OCTA is limited by its inability to detect vascular leakage and the fact that it still cannot visualize the entire periphery. To avoid erroneous interpretation of OCTA images, the examiner must also be aware of the artifacts that may arise in data acquisition or image calculation [4,5]. The aim of this article is to describe the current routine use of OCTA in patients with neovascular age-related macular degeneration (AMD) and thus to show the practical utility of this means of diagnosis.

## 2. Age-Related Macular Degeneration

AMD is a progressive eye condition and a significant cause of irreversible blindness among older individuals worldwide. Over the past decade, the disease has become more evident due to the exponential increase in life expectancy globally. In 2020, approximately 200 million individuals worldwide were affected by AMD. By 2040, it is estimated that around 288 million people will suffer from the disease. This will pose significant economic and social burdens on healthcare systems and greatly affect the quality of life for elderly populations. AMD contributes to almost 9% of global blindness cases [6]. AMD leads to pathological changes in the deeper layers of the macula, resulting in impaired central vision. The presence of retinal deposits called drusen is a key indicator of dry AMD and often marks the initial stage of the disease.

Major risk factors for AMD comprise getting older, smoking, high blood pressure, atherosclerosis, and harm from light exposure leading to oxidative stress [7]. Additionally, genetic factors play a role, including mutations in genes associated with the complement system, as well as irregularities in lipid metabolism, angiogenesis, and the extracellular matrix pathways [8,9].

Drusen, which are yellowish deposits, form between the RPE and the inner collagen layer of Bruch’s membrane (BM). These deposits hinder the flow of essential nutrients and waste products between the choriocapillaris and the neural retina. Drusen consists of various substances, including plasma proteins, apolipoprotein E, cholesterol-rich lipids, polysaccharides, glycoproteins, and plasma amyloid P. Studies have shown that these substances deactivate the complement system and contribute to the formation of membrane attack complexes [10]. In the late stage of AMD, the thickening of BM, accumulation of metabolic products, oxidative stress, and inflammation with the release of angiogenetic factors can lead to the development of macular neovascularizations (MNV) beneath and within the retina [11,12]. This neovascular form of AMD (nAMD) progresses swiftly and aggressively, so rapid diagnosis and treatment are essential to optimize the preservation of visual acuity [13]. The other late stage of AMD is geographic atrophy (GA), in which there is increasing atrophy of the outer retinal layers, photoreceptors, and the RPE.

## 3. Diagnosis of AMD

A fundus examination in mydriasis should be performed. During this examination, the presence of drusen deposits, changes in pigmentation, GA, hemorrhage, fluid leakage, scarring, and fibrosis are assessed to diagnose AMD. The size, distribution, and quantity of drusen are carefully considered. A comprehensive eye examination is conducted to rule out any other concurrent eye disorders. While the examination provides valuable insights into disease staging, the integration of various imaging techniques has become essential for confirming findings and guiding treatment decisions.

Currently, the diagnosis of nAMD prompts invasive treatment with intravitreal administration of anti-VEGF substances or bispecific antibodies [13]. The intravitreal application is potentially associated with risks such as the occurrence of eye-threatening endophthalmitis, so high sensitivity and specificity in the diagnosis of nAMD are important.

Fluorescein angiography (FA) has been the gold standard method for identifying MNV in AMD. This involves injecting fluorescein dye into a patient’s vein, followed by capturing images of the chorioretinal circulation over several minutes. FA, although invasive, can detect leakage from different types of MNV lesions. The advent of optical coherence tomography (OCT) has revolutionized the understanding and management of AMD. OCT, a noninvasive technology provides detailed cross-sectional images of the retinal layers, allowing precise assessment of disease stage and MNV activity [14]. By using a light source, OCT enables the identification of affected layers in AMD. Additionally, OCT can longitudinally monitor treatment response and guide further management by revealing fluid accumulation in the retina. OCT angiography (OCTA) is a newer technique that offers enhanced visualization of the choroidal vascular network without the need for dye injection. OCTA facilitates a better understanding of microvascular changes in nAMD and allows early detection of neovascularization leading to more proactive surveillance and timely intervention [15].

**Figure 1:**
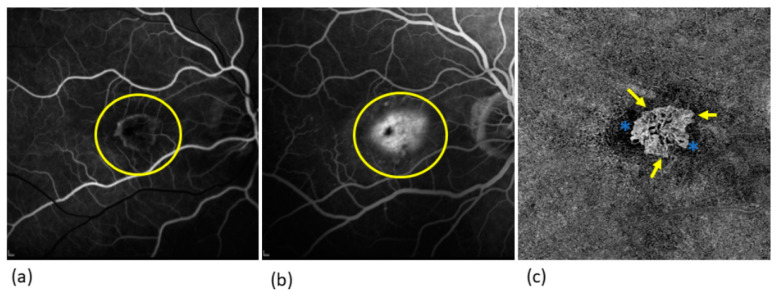
FAG and OCTA image of a type 2 MNV in comparison: (**a**) Early phase of FAG: here the structure of the MNV is very delicate (yellow circle); (**b**) late phase of FAG: the leakage of fluorescein from the MNV results in the typical hyperfluorescence with an increase in the size of the lesion (yellow circle); (**c**) OCTA: the MNV can be seen in greater detail with its vascular structure. The MNV has a prominent central vessel from which the smaller vessels arise laterally, which is known as the medusa shape. In the border area as activity parameters vessel loops, arcades, and capillaries (yellow arrows), and a surrounding halo (blue stars). The MNV is well-demarcated from the surrounding tissue.

A number of studies have compared OCTA with the FA. The sensitivity of OCTA for the detection of an MNV in nAMD has been reported as 66.7–86.5%, and the specificity as 67.6–100% [16,17,18,19]. A few studies have compared measurements of the area of MNV by OCTA and FA. In this regard, it is particularly important to note that OCTA visualizes vessels only indirectly by means of detecting the velocity and direction of blood flow, which is not necessarily equivalent to the presence of blood vessels (Figure 1) [20]. Additionally, correct segmentation is essential for viewing MNV, ensuring it is fully captured and not partially or entirely excluded. It is important to note that type 1 and type 2 MNV originate in the choriocapillaris and grow towards the RPE. Type 2 MNV further penetrates the RPE into the subretinal space. In contrast, type 3 MNV originates in the deep retinal vascular plexus and grows in the opposite direction towards the RPE [15].

## 4. The Morphology of MNV in nAMD

Costanzo et al. found that the MNV area was 25% lower with OCTA than with FA. This may be because the blurring of MNV margins by leakage of fluorescein makes the area appear larger [21]. In contrast, Lindner et al. described comparable areas of MNV on OCTA and FA; nevertheless, it was striking that the interexaminer agreement was significantly greater for OCTA than for FA (0.884 vs. 0.636), indicating a more precise delineation of MNV from the surrounding tissues [22].

**Figure 2:**
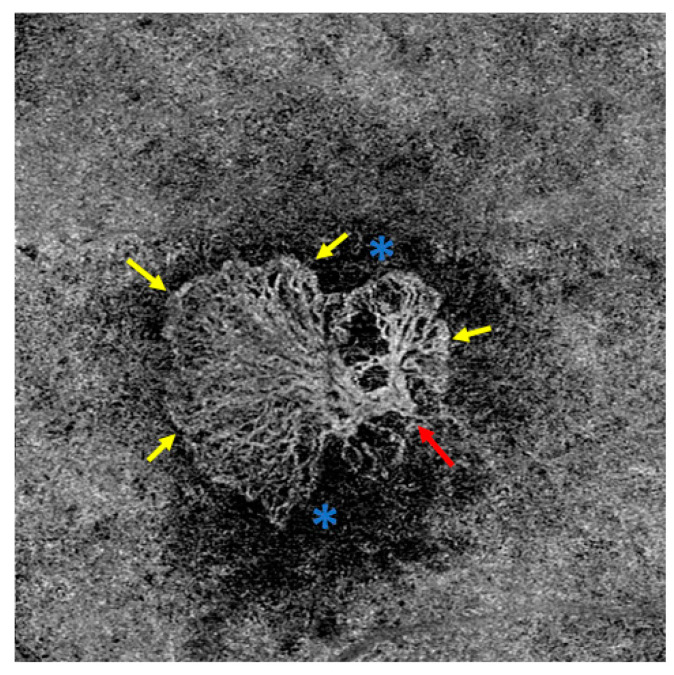
Sea-fan form MNV in the OCTA with prominent feeding cutaneous vessel (red arrow) and smaller vessels with capillaries branching off in one direction. The MNV is well demarcated from the surrounding tissue, in the marginal area of the MNV there are vascular loops, peripheral vascular arcades, and a capillary network (yellow arrows). The MNV is surrounded by a dark halo (blue stars). These features all represent activity criteria.

**Figure 3:**
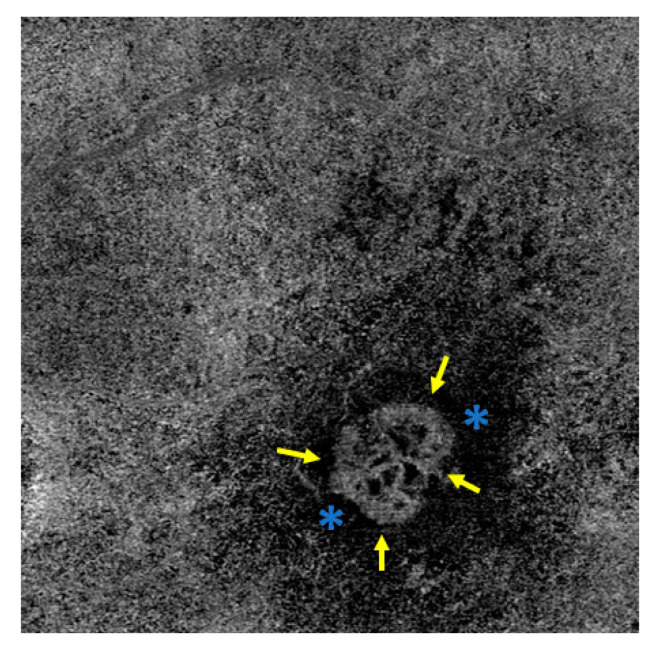
Visualization of a glomerulus form MNV in OCT with interconnected vessels. In the border area as activity parameters vessel loops, arcades, capillaries (yellow arrows), and a surrounding halo (blue stars). The MNV is well-demarcated from the surrounding tissue.

El Ameen et al. and Kuehlewein et al. were the first to provide morphological evaluations of MNV in OCTA [23,24]. In articles published in 2015, they described the following forms:

- Medusa form: one prominent central vessel with smaller vessels radiating in all directions (Figure 1);
- Sea-fan form: one prominent central vessel with smaller vessels all radiating in the same plane (Figure 2);
- Glomerulus form: a globular structure of interconnected vessels (Figure 3).

**Figure 4:**
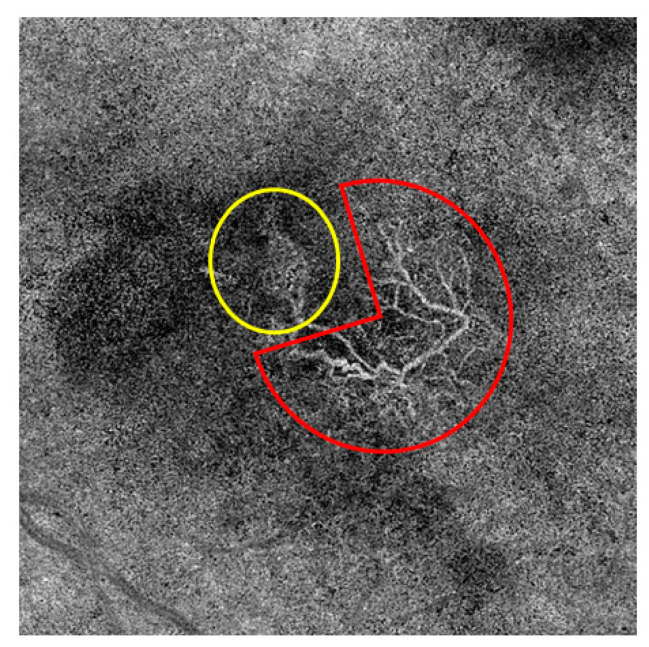
MNV visualized using OCTA: In the yellow area, you can see a part of the MNV that corresponds to a dense network with many capillaries and vessel crossings. In the red area, the MNV part is depicted with a loose network configuration, consisting of large mature vessels with few vessel crossings.

They also mentioned an undefined form of MNV in which no clear vascular pattern could be defined or no MNV vessels at all could be discerned. These authors were unable to demonstrate any association of MNV form with pigment epithelial detachment (PED), subretinal hyperreflective material (SHRM), atrophy of the retinal pigment epithelium (RPE), or the number of intravitreal anti-VEGF treatments given [23]. Sulzbacher et al. categorized the vascular structure of MNV as a dense-net configuration, a loose-net configuration, a mixed type (Figure 4), or an unidentifiable pattern and investigated a total of 88 eyes that either had treatment-naive nAMD or had received anti-VEGF therapy for a number of years. They found no significant differences among the categories with regard to vision, PED, intraretinal fluid (IRF), or subretinal fluid (SRF), but showed that disease duration was significantly longer for the loose than for the dense vascular structure (median 4.3 years vs. 2.0 years; *p* < 0.009). Moreover, the mixed structure was associated with a significantly greater median disease duration (4.7 years) than the unidentifiable pattern (1.1 years, the shortest median duration) [25]. This suggests that the structure of MNVs changes over time in that they develop more prominent vessels with less branching, leading to good visualization by OCTA devices. In contrast, early-stage MNVs, with large numbers of small branching vessels and capillaries, are more difficult to visualize [26]. The impact of anti-VEGF treatment on the long-term reorganization of the vascular structure of MNV has not yet been fully clarified. Short-term effects of anti-VEGF therapy, however, can readily be discerned. For example, Pilotto et al. showed that the density of fine vessels was reduced in 75% of eyes, whereas that of larger vessels remained unchanged in two-thirds of cases [26]. It must be noted that this does not necessarily mean that the fine vessels disappear as the result of the anti-VEGF treatment; rather, they may be undetectable due to temporary cessation or pronounced reduction of blood flow, only to be visualized again after reactivation.

Furthermore, OCTA may help to differentiate the various subtypes of nAMD. Type 1 MNV arises from the choroid and occupies the space between Bruch’s membrane and the RPE. Type 2 MNV also originates from the choroid but penetrates the RPE and infiltrates the subretinal space. Some MNV spread both under the RPE and subretinally; these are designated mixed type 1/2. In contrast, type 3 MNV has its origin in the deep retinal vascular plexus and grows towards the choroid. These different sites of pathological vessels can be precisely localized on OCTA by the selection of segmentation or the flow signal in the B scan [15]. Studies have shown variations in the vascular architecture of the MNV subtypes. Nakano et al. demonstrated that type 1 MNV has a lower density of vessel junctions than type 2 MNV [27]. A previous study by our group showed a high degree of similarity in vascular morphology between type 1 and type 2 MNV, but type 2 MNV displayed significantly smaller area (*p* < 0.00001), lower fractal dimension (*p* < 0.00001), and higher vessel junction density (MNV 1: *p* < 0.05; MNV 21: *p* < 0.005) [28].

## 5. Activity Parameters and Biomarkers

Decisions on treatment monitoring and repetition of anti-VEGF therapy are currently based on IRF, SRF, PED, and the occurrence of bleeding, all of which are complications of MNV growth. The sole evidence of an MNV in OCTA does not yet constitute an indication for treatment with anti-VEGF preparations. It may be a non-exudative MNV that may have been present for many years and for which therapy would not provide any positive benefit. Additionally, MNV with exudation can still be detectable after anti-VEGF therapy in a temporarily inactive stage, but without exudation, they do not constitute an indication for treatment. It would be useful to be able to evaluate the activity of MNV vessels reliably by means of OCTA, enabling detection of activity and thus initiation of treatment before the retinal cells are damaged further [29].

Coscas et al. defined five criteria of MNV activity on OCTA:

1. Clear delineation of the MNV from the surrounding tissues (Figure 1 Figure 2 Figure 3);
2. A high-density capillary network (Figure 1 Figure 2 Figure 3);
3. Anastomoses/vascular loops (Figure 1 Figure 2 Figure 3);
4. Peripheral vascular arcades (Figure 1 Figure 2 Figure 3);
5. A hypointensive halo around the MNV (Figure 1 Figure 2 Figure 3).

The authors applied these criteria of activity to 80 eyes of patients with nAMD. In those in which three or more criteria were fulfilled, there was 94.9% correspondence with FA and SD-OCT. Of the patients, 90.5% who met less than three of the activity criteria also showed no activity criteria on FA and SD-OCT [30]. Cho et al. investigated the vascular structure of MNV in patients with nAMD during the treatment-free interval and, in partial agreement with Coscas et al., identified certain characteristics as advanced signs of new exudation: 53.3% showed minuscule branched vessels; 40%, anastomotic loops; 44.4%, peripheral vascular arcades; and 35.6%, a surrounding halo. The mean time from occurrence of these vessel changes to exudation was 2.3 ± 2.0 months [31]. Hikichi et al. observed patients with nAMD during the inactive stage after anti-VEGF treatment and noted a significant increase in MNV size in those who later experienced further exudation, in contrast to patients who remained inactive (*p* < 0.001) [32]. We also showed in a previous study that anti-VEGF treatment was followed by significant reductions in MNV area, MNV total vessel length, MNV vessel segments, and fractal dimension that correlated with a decrease in central retinal thickness (*p* < 0.00001) [33]. While some authors have also observed a reduction in MNV area during anti-VEGF treatment [25,34], other studies found no change in size; these, however, were explicitly restricted to type 3 MNV [35]. In contrast, over a period of a year of anti-VEGF treatment, the majority of cases show progressive MNV growth [36]. Greater MNV area on OCTA is also correlated with poorer baseline vision, as well as with less improvement of vision in the course of treatment [37,38]. This corresponds with the results of the large randomized controlled trials MARINA [39], ANCHOR [40], CATT [41], and VIEW [42], which also showed a lesser increase in vision after anti-VEGF treatment in patients with greater MNV area.

It would be particularly advantageous to have biomarkers that predict the disease course, the need for treatment, and the functional outcome in a given patient at the time of diagnosis. It would also be helpful, with regard to the development of new drugs, to have some indication of which individual patient will benefit the most from which medication. Arrigo et al. determined that the degree of tortuosity of MNV vessels at the time of diagnosis correlates significantly with the visual outcome [43]. They also found that patients with a high tortuosity index exhibited less exudation, but over 30% developed external retinal atrophy and thus irreversible vision loss [44].

There are numerous ways of describing MNV mathematically. The diverse techniques all depend on the device used and on the algorithms utilized for image calculation, so that blood vessel presentation can vary substantially between different devices and different sizes of images [45,46]. This must always be borne in mind and is one of the reasons why no consensus has yet been achieved regarding the importance of individual parameters.

Another phenomenon described by a number of authors using OCTA is a dark halo around the vast majority of type 2 MNV and 51% of type 1 MNV [24,47]. Rispoli et al. examined the change in the MNV area and halo area during anti-VEGF treatment and showed that both MNV and halo decreased significantly (*p* < 0.001 in each case) [48]. Viggiano et al. demonstrated similar results and described reperfusion of the halo after anti-VEGF treatment [49]. Fossataro et al., comparing the area of the halo on OCTA and indocyanine green angiography (ICGA), found significantly greater extension on OCTA (*p* < 0.001) with slight agreement between ICGA halo and OCTA halo (intraclass coefficient 0.397) [50]. The authors therefore discussed the possible role of the halo as a parameter of activity during treatment monitoring. However, the halo phenomenon is still not fully clarified: the blood flow in this area may be undetectable due to slowness or turbulence; compression of the choriocapillaris by the MNV or by exudation may be the cause; or an artifact may be at fault.

Artificial intelligence (AI) offers considerable advantages in image analysis, and its application to retinal diagnosis has been investigated in a number of studies. Wang et al. developed an algorithm for the detection of MNV on OCTA images and achieved a sensitivity of 100% and specificity of 95% [51]. Another study, which also included OCTA scans of neovascularizations (NV) in diseases other than nAMD and featured automated analysis by an algorithm, attained 95% rates of both sensitivity and specificity for NV [52]. AI can also extract specific information for the assessment of activity from OCTA images. In this regard, an algorithm developed by Jin et al. for activity assessment by analysis of SD-OCT and OCTA images from patients with nAMD showed that the best result, with an area under the curve of 0.9928, was achieved when the algorithms inspected the OCTA images primarily and the SD-OCT images only secondarily [53]. The AI generates so-called heat maps, which highlight areas on the OCTA images that were particularly important for the AI algorithm’s decision-making process. However, it is not possible to fully trace the exact image information that led the AI algorithm to its conclusion. OCTA will therefore have a particularly prominent role in future multimodal image analysis.

## 6. Non-Exudative MNV

Studies have found that a very high proportion of patients with nAMD in one eye (42–58%) go on to develop nAMD in their other eye within 5 years [54,55,56]. We know from the AREDS study that large drusen and pigment epithelial lesions as well as the MNV in the fellow eye represent a high risk of conversion to wet AMD [56]. OCTA is the first modality with the capacity to detect MNV that are not yet exudative [57]. Moreover, it has been demonstrated that patients with subclinical MNV are much more likely to develop exudation later. In an observational case series, de Oliveira Dias et al. found that patients with a non-exudative MNV had a 15.2-fold risk of developing exudation within 1 year [58]. Yang et al. followed up patients with intermediate AMD for 2 years: 13.2% already had a subclinical MNV at the beginning of the observation period, and 8.9% developed a subclinical MNV during follow-up. Over the 2 years, the relative risk of developing exudation was 13.2 times higher with a subclinical MNV than without [59]. Bailey et al. also used OCTA to observe the dry fellow eye of patients with nAMD over a period of 2 years and discovered that detection of a non-exudative MNV was associated with an 18.1-fold risk of developing exudation (*p* < 0.0001) [60]. A non-exudative MNV is often found by OCTA in the fellow eye of patients with nAMD and carries a high risk of later exudation. These patients should be warned to be alert for changes in vision and given the benefit of close monitoring so that the necessary treatment can be initiated promptly.

OCTA can also be helpful in detecting non-exudative MNV in patients with geographic atrophy (GA); indeed, it is superior to dye-based angiography and SD-OCT in this regard [61]. In the pivotal study of pegcetacoplan for the treatment of GA, exudation occurred in a striking number of treated patients with MNV relative to the sham control group [62]. Moreover, the rate was higher in the patients who were treated every month than in those treated every 2 months. It has not yet been established whether pre-existing MNVs undergo conversion to exudation or whether new MNVs develop. OCTA could play an important part in clarifying this situation and better estimating the individual risk of MNV development in patients with GA on treatment with pegcetacoplan.

## 7. Summary

In contrast to FA, OCTA is a non-invasive method of visualizing retinal and choroidal vessels. It is a comfortable procedure that offers highly reproducible imaging of physiological and pathological vascularization patterns and is thus often a useful component of the routine diagnostic work-up. When interpreting OCTA scans, one must always keep in mind that the blood vessels depicted are detected on the basis of blood flow in a defined time window and no leakage phenomena can occur. Very fast, very slow, or turbulent flow therefore often goes undetected. The segmentation should be checked by means of a B-scan. The potential existence of artifacts in the images must also be well understood in order to avoid misinterpretation. The segmentation lines in the B-scan should also always be taken into consideration to enable the classification of the image contents and recognize any segmentation errors. As long as these stipulations are observed, the OCTA images almost always yield important data on the perfusion status of the retina and choroid that contribute to establishing the diagnosis.

OCTA is an attractive option for the imaging of MNV in nAMD, offering high sensitivity. Specific vessel characteristics and changes in vasculature can be used as parameters of activity. In addition, OCTA enables better determination of the individual risk of conversion to nAMD in patients with intermediate AMD. The technical advancement of OCTA devices with higher resolution, fewer artifacts, and more accurate detection of blood flow will improve analyses in the future. Additionally, there is hope that AI can be used to further analyze the large datasets and identify additional biomarkers. There are also grounds for optimism that the detailed information on MNV vasculature provided by OCTA may enable the identification of new biomarkers for individualized prediction of the course of the disease, the need for treatment, and the functional outcome. This could, particularly in view of the development of novel drugs, contribute to more personalized treatment monitoring and decision-making. Further large studies need to be conducted for this purpose. Ideally, OCTA should be among the imaging procedures performed in future pivotal studies.

## Data Availability

The results presented are provided with references.
